# Adverse childhood experiences and depression among women in rural Pakistan

**DOI:** 10.1186/s12889-021-10409-4

**Published:** 2021-02-25

**Authors:** Katherine LeMasters, Lisa M. Bates, Esther O. Chung, John A. Gallis, Ashley Hagaman, Elissa Scherer, Siham Sikander, Brooke S. Staley, Lauren C. Zalla, Paul N. Zivich, Joanna Maselko

**Affiliations:** 1 Department of Epidemiology, Gillings School of Global Public Health, University of North Carolina at Chapel Hill, McGavran-Greenberg Hall, CB# 7435, Chapel Hill, NC 27599 USA; 2 Carolina Population Center, Chapel Hill, North Carolina USA; 3 Department of Epidemiology,Mailman School of Public Health, Columbia University Medical Center, 722 West 168th Street, New York, NY 10032 USA; 4 Department of Biostatistics and Bioinformatics, Duke University, 2424 Erwin Road, Durham, NC 27705 USA; 5 Duke Global Health Institute, Duke University, Durham, North Carolina USA; 6 Department of Social and Behavioral Sciences, Yale School of Public Health, Yale University, 135 College St., Suite 200, Room 230, New Haven, CT 06510 USA; 7 RTI International, 3040 E Cornwallis Rd, Durham, NC 27709 USA; 8 Human Development Research Foundation, H 06, Street 55, Sector F-7/4, Islamabad, 44000 Pakistan; 9 Health Services Academy, Islamabad, Pakistan

**Keywords:** Perinatal depression, maternal depression, Pakistan, adverse childhood experiences

## Abstract

**Background:**

Adverse Childhood Experiences (ACEs) are a common pathway to adult depression. This pathway is particularly important during the perinatal period when women are at an elevated risk for depression. However, this relationship has not been explored in South Asia. This study estimates the association between ACEs and women’s (*N* = 889) depression at 36 months postpartum in rural Pakistan.

**Method:**

Data come from the Bachpan Cohort study. To capture ACEs, an adapted version of the ACE-International Questionnaire was used. Women’s depression was measured using both major depressive episodes (MDE) and depressive symptom severity. To assess the relationship between ACEs and depression, log-Poisson models were used for MDE and linear regression models for symptom severity.

**Results:**

The majority (58%) of women experienced at least one ACE domain, most commonly home violence (38.3%), followed by neglect (20.1%). Women experiencing four or more ACEs had the most pronounced elevation of symptom severity (β = 3.90; 95% CL = 2.13, 5.67) and MDE (PR = 2.43; 95% CL = 1.37, 4.32). Symptom severity (β = 2.88; 95% CL = 1.46, 4.31), and MDE (PR = 2.01; 95% CL = 1.27, 3.18) were greater for those experiencing community violence or family distress (β = 2.04; 95%; CL = 0.83, 3.25) (PR = 1.77; 95% CL = 1.12, 2.79).

**Conclusions:**

Findings suggest that ACEs are substantively distinct and have unique relationships to depression. They signal a need to address women’s ACEs as part of perinatal mental health interventions and highlight women’s lifelong experiences as important factors to understanding current mental health.

**Trial registration:**

NCT02111915. Registered 11 April 2014. NCT02658994. Registered 22 January 2016. Both trials were prospectively registered.

## Background

Women are at an elevated risk for depression during the perinatal period, which can impair a woman’s health, increase her risk of suicide, and impact her child’s growth and development [1–3]. Additionally, women experiencing perinatal depression are at risk for recurrent depressive episodes, which cause further deleterious outcomes for maternal and child health [3]. In low- and middle-income countries (LMIC), the pooled prevalence of postpartum depression (beginning in pregnancy up to 1 year postpartum) is estimated to be 19.8% [4]. The highest burden of postpartum depression is in LMIC, exacerbating economic and social inequalities and making depression a global health priority [1, 2].

Adverse childhood experiences (ACEs), measured by the ACE questionnaire and classified as abuse, neglect, household dysfunction, and community disfunction, are a common pathway to long-term social, emotional, and cognitive impairments, including depression [1, 5]. Globally, the majority of individuals (57%) experience at least one ACE [6]. The prevalence of ACEs also varies by context, with LMIC settings consistently reporting higher rates than high-income countries [7]. Prior research has found ACEs to be related to prenatal depressive symptoms and postpartum depression [6, 8]. Additionally, there is a dose-response relationship between how many ACEs a woman experiences and the likelihood of perinatal depressive symptoms [9].

However, there is a lack of consensus about which types of ACEs (i.e., abuse; household dysfunction) are related to women’s depression, as this relationship likely varies by context [10]. For instance, one study in the United States (US) found ACE score and maltreatment to be associated with prenatal depression but found no relationship between household dysfunction and depression [11]. However, a study in Canada found household dysfunction and abuse to be related to maternal depression [10]. Lastly, most research has measured this relationship during pregnancy and 1 year postpartum, so it is not understood if ACEs are related to depression beyond this high-risk window.

Further, ACEs research, including research assessing relationships between ACEs and women’s depression in adulthood is concentrated in high-income countries [1, 6]. ACEs may manifest differently in LMIC due to different norms and resources. The ACE-International Questionnaire (ACE-IQ), designed and adapted to study ACEs outside the US, adds items to assess the experience of peer violence, exposure to collective violence, and witnessing community violence, which are more commonly experienced in LMIC [12].

In Pakistan, a LMIC and the setting of the current study, research has linked the ACE-IQ to physical and mental health outcomes among a student population in an urban setting [13, 14]. However, the impact on adult rural women’s mental health of ACEs exposure, overall or by type, has not been investigated. Additionally, the prevalence of postpartum depression in Pakistan is estimated to be higher than other countries in South Asia and than most LMIC at 28–36% [4, 15, 16]. Specific aspects of the social context of rural Pakistan may be particularly relevant to understanding this relationship among adult women. For example, over two-thirds of rural families live in multigeneration or extended family homes [17, 18], 15% of women have completed secondary education, the fertility rate is 3.9, and over 30% of families live in poverty [19, 20]. Women’s exposure to marital intimate partner violence is also quite prevalent, as it is elsewhere in South Asia [21, 22].

This study estimates the overall relationship between ACEs and women’s depression at 36 months postpartum in Pakistan and assesses which ACE domains are related to women’s depression. As depression can impair women’s well-being, understanding underlying risk factors (i.e., ACEs) can lead to developing interventions to improve mental health [23]. Additionally, by investigating this relationship in a LMIC, global health practitioners can better target mental health interventions to those at highest risk of depression.

## Methods

### Study design and participants

The Bachpan Cohort study is located in rural Pakistan in the north of the Punjab Province in the rural sub-district of Kallar Syedan [24]. It consists of a cluster randomized controlled trial nested in a longitudinal birth cohort. The objective of Bachpan is to evaluate the impact of a peer-delivered, community-based intervention on maternal depression and child development [24]. In 40 village clusters, from 2014 to 2016, all women in their third trimester were invited to be screened for depression using the Patient Health Questionnaire (PHQ-9) [24]. In each village, women who screened positive for depression (i.e., had a PHQ-9 score ≥ 10) were eligible for participation in the trial and follow-up as part of the cohort, and one of every three women with a PHQ-9 score of < 10 were eligible to participate in the cohort as a non-depressed reference group, resulting in roughly equal numbers of depressed to non-depressed women at baseline [24].

Assessments occurred at six time points: in women’s third trimester and at three, six, 12, 24, and 36 months postpartum. This analysis utilized data collected at pregnancy (baseline) and 36 months postpartum. As questions on ACEs were included in the 36-month questionnaire, the current analysis uses the depression outcome also from the 36 month wave as well. Of the 1154 women enrolled in their third trimester, 265 were lost to follow-up by 36 months, resulting in 889 women in our analytic sample.

### Measures

#### Outcomes

The primary outcomes of interest were major depressive episode (MDE) and depressive symptom severity. MDE was evaluated with the Urdu version of the Structured Clinical Interview for the Diagnostic and Statistical Manual of Mental Disorders’ Module for Current MDE (SCID) [25]. Depressive symptom severity was evaluated using The PHQ-9, which has been extensively used as a screening tool in this study setting and has an acceptable criterion validity and reliability for this population [26]. The PHQ-9 has nine items, each with a score from zero to three; individuals can thus receive a maximum score of 27. A score ≥ 10 is commonly used to indicate symptoms reaching a clinically significant level and was treated as a continuous variable in this analysis [26].

#### Exposure

ACEs were measured through the 12-item ACE-IQ, which has been validated in international settings (Appendix Table 4) [27]. The ACE-IQ is a retrospective report of women’s experiences prior to age of 18. The ACE-IQ was adapted by removing the sexual abuse questions due to potential risks to the respondent and the belief that underreporting would be high. The ACE-IQ was also translated into Urdu. We created a summed score of women’s experiences, a categorical variable indicating the number of experiences reported (0, 1, 2, 3, 4+), and indicators for each of the following domains: neglect (emotional neglect; physical neglect), family psychological distress (alcohol and/or drug abuser in the household; incarcerated household member; someone depressed, mentally ill, institutionalized or suicidal), home violence (physical abuse; emotional abuse; household member treated violently), and community violence (bullying; community violence; collective violence). Each indicator was coded as ‘yes’ if a woman experienced any of the ACEs within the domain and ‘do not remember’ was coded as ‘no.’ Twenty of the 889 women had responses originally coded as ‘do not remember.’

#### Confounders

Confounders in these models were selected for consideration using a Directed Acyclic Graph (DAG). These were assessed at baseline and included age, natal family’s history of mental illness, and education. In this context, education is a marker for childhood socioeconomic status (SES). Accordingly, years of schooling was recoded in this analysis as a binary indicator (at least primary attainment versus less) because receiving a primary education can approximate family SES given educational expenses [28]. Age was coded linearly and family history of mental illness was assessed with a binary indicator.

### Statistical analysis

Given unequal probabilities of selection into the study, sampling weights were used to represent the population of pregnant women in the area. Specifically, non-depressed women were up-weighted to account for their sub-sampling during recruitment when all women were screened for depression [28]. Cluster-specific weights were created for the non-depressed women to match their sampling fraction. All non-depressed women in a given cluster were weighted by the same value, the inverse of the proportion of non-depressed women in the sample of women screened for depression that were subsequently enrolled. This was in contrast to the depressed women who were all invited to participate and received a weight of one. These cluster-specific sampling weights were applied to all analyses and statistics.

To estimate the relationship between ACEs and SCID, we used log-Poisson models to estimate prevalence ratios [29]. For ACEs and PHQ-9, we used linear regression. In all models, village was taken into account by using cluster robust standard errors. In addition to aforementioned confounders, all models were adjusted for trial arm and assessor. To account for potentially informative loss-to-follow-up by observed characteristics, stabilized inverse probability of censoring weights (IPCW) were calculated as:

$$ IPCW=\frac{\Pr \left(C=0\ \right)}{\Pr \left(C=0\ |W\right)} $$

where *C* indicates participants being lost-to-follow-up before 36 months, *W* is a set of baseline confounders determined a priori (age, natal family’s history of mental illness, education) and baseline predictors of censoring, determined by *p* < 0.10 (crowding, grandmother co-residence, number of living children, SCID, and trial arm), with asset score included to increase precision (*p* = 0.11) (Appendix Table 5) [30]. IPCW account for informative loss-to-follow-up by observed variables through re-weighting individuals with completed follow-up to ‘stand-in’ for those who were lost to follow-up [31]. Sampling weights and IPCW were multiplied together to obtain the final weight [32]. Robust variances were similarly used to account for clustering and the additional weights. Analyses were conducted using Stata 16.

## Results

### Descriptive statistics

Our sample comprised 889 mothers as 265 were lost to follow-up by 36 months. After applying population-representative weights and IPCW, women were, on average, 26.7 years old (Tables 1-2). The majority had over a fifth-grade education (69.5%) and 10% lived with someone with a mental illness growing up. At 36 months postpartum, 12.4% had a MDE and 16.8% had a PHQ-9 score above the cutoff (PHQ-9 ≥ 10) for moderate depressive symptoms. Fifty-eight percent had experienced at least one ACE. Among the 12 ACE categories, emotional abuse (31.9%), physical abuse (22.5%), and emotional neglect (15.6%) were the most common. Regarding ACE domains, over a third (38.3%) were exposed to home violence and one-fifth had experienced emotional or physical neglect (20.1%). In comparison, family psychological distress and community violence were less common (15.8 and 6.6%, respectively).

**Table 1:** ACE Characteristics^a^, Bachpan Cohort, Pakistan, *N* = 889^b^

Descriptor	N	%
**Neglect**		
(1) Emotional Neglect	134	15.54
(2) Physical Neglect	49	5.71
**Family Psychological Distress**		
(3) Alcohol and/or drug abuser in the household	24	2.57
(4) Incarcerated household member	16	1.55
(5) Someone chronically depressed, mentally ill, institutionalized or suicidal	21	2.14
(6) Divorce	97	10.97
**Home Violence**		
(7) Physical Abuse	209	23.28
(8) Emotional Abuse	294	32.39
(9) Household member treated violently	130	14.92
**Community Violence**		
(10) Bullying	12	1.25
(11) Community Violence	60	6.56
(12) Collective Violence	4	0.45
**Domains**		
Neglect	173	20.04
Family Psychological Distress	143	15.81
Home Violence	349	38.94
Community Violence	64	6.95
**Number of ACEs**		
0 ACEs	369	41.25
1 ACE	239	27.74
2 ACEs	139	14.98
3 ACEs	82	9.36
4+ ACEs	60	6.67
Total Number of ACEs	**Mean**	**SD**
	1.17	1.39

^a^All characteristics are based on mother’s recall at 36 months

^b^N’s are unweighted while %, Mean, and SD are weighted by sampling weights and Inverse Probability of Censoring Weights. All items were assessed at 36 months

**Table 2:** Descriptive Characteristics^a^, Bachpan Cohort, Pakistan, *N* = 889^b^

	Mean	SD
Age	26.58	4.46
	**N**	**%**
Education >5th grade	590	68.87
Mental Health Issues in Natal Family	96	9.59
SCID (36 months)	124	12.35
	**Mean**	**SD**
PHQ-9 (36 months)	4.34	5.25

^a^All are baseline (prenatal) descriptive characteristics except where otherwise indicated

^b^ N’s are unweighted while %, Mean, and SD are weighted by sampling weights and Inverse Probability of Censoring Weights. Items were assessed at baseline unless otherwise indicated

### Symptom severity and MDE diagnosis

**Fig. 1:**
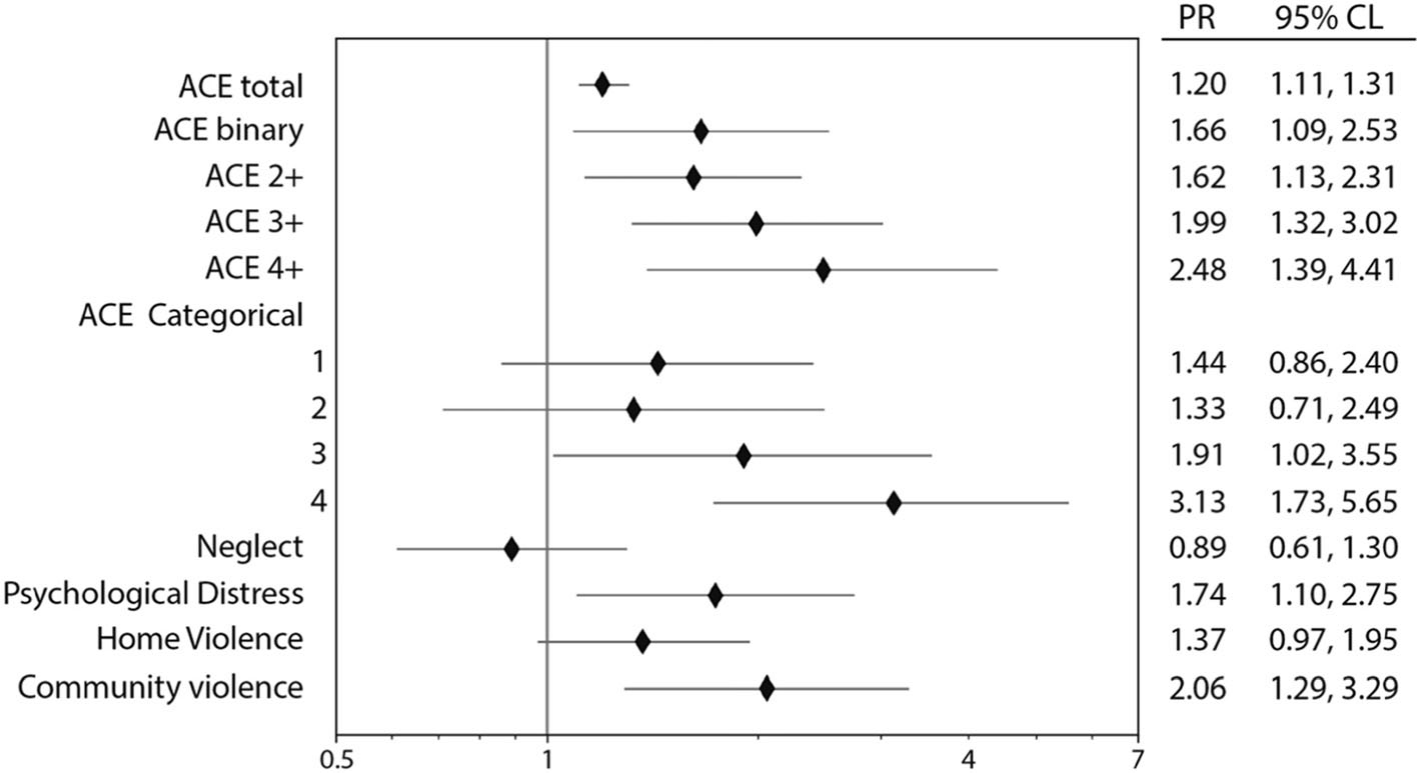
Maternal ACEs and Depression (SCID) at 36 Months Postpartum, Bachpan Cohort, Pakistan, *N* = 889. Models account for clustering using cluster robust standard errors, and used weights which were a combination of sample weights and inverse probability of censoring weights. All models were adjusted for age, education, and mental health problems in natal family as confounders, and adjusted for trial arm and assessor. Abbreviations: PR – Prevalence ratio; B – Estimate; CL – Confidence Limit; ACE – Adverse Childhood Experiences For ACE Categorical, 0 ACEs is the reference level

**Fig. 2:**
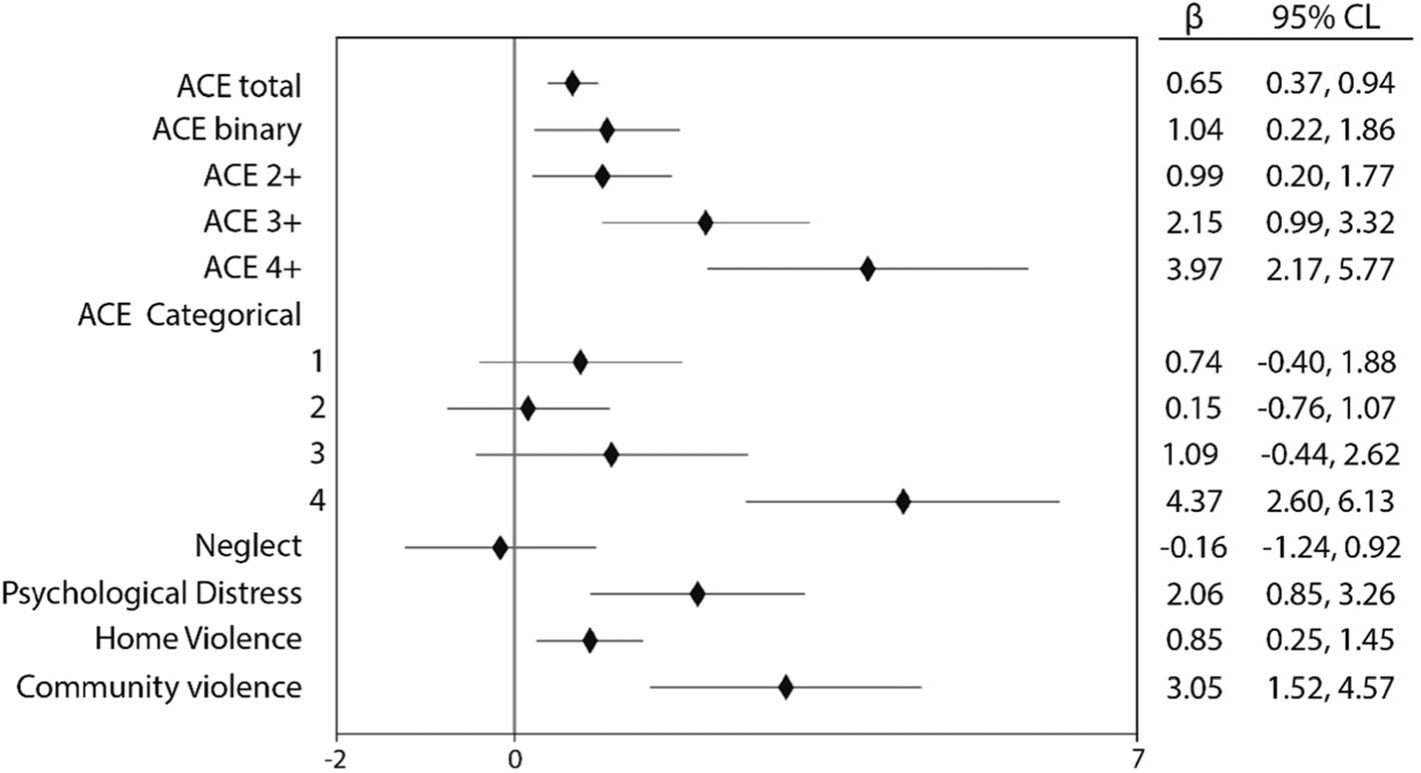
Maternal ACEs and Depression (PHQ-9) at 36 Months Postpartum, Bachpan Cohort, Pakistan, *N* = 889. Models account for clustering using cluster robust standard errors, and used weights which were a combination of sample weights and inverse probability of censoring weights. All models were adjusted for age, education, and mental health problems in natal family as confounders, and adjusted for trial arm and assessor. Abbreviations: B – Estimate; CL – Confidence Limit; ACE – Adverse Childhood Experiences. For ACE Categorical, 0 ACEs is the reference level

Total ACE score was associated with poor mental health (Table 3; Figs. 1 and 2). Exposure to ACEs (Model 1) was positively related to both MDE (Prevalence ratio [PR] = 1.20; 95% Confidence Limit [CL] = 1.11,1.31) and symptom severity (Estimate (β) = 0.65; 95% CL = 0.37,0.94). While there was not a clear step-wise trend between incremental exposure to ACEs and depression (Models 2–5), the experience of four or more ACEs (Model 6) was related to a higher prevalence of MDE (PR = 3.13; 95% CL = 1.73,5.65) and stronger symptom severity (β = 4.37; 95% CL = 2.60,6.13) compared to those experiencing no ACEs. We found no relationship between neglect (Model 7) and MDE (PR = 0.89; 95% CL = 0.61,1.30) or symptom severity (β = − 0.16; 95% CL = -1.24,0.92). Family psychological distress (Model 8) was associated with MDE (PR = 1.74; 95% CL = 1.10,2.75) and symptom severity (β = 2.06; 95% CL = 0.85,3.26). Home violence (Model 9) was associated with both MDE (PR = 1.37; 95% CL = 0.97,1.95) and symptom severity (β = 0.85; 95% CL = 0.51,1.45). Lastly, experiencing community violence (Model 10) was associated with poor mental health for both MDE (PR = 2.06; 95% CL = 1.29,3.29) and symptom severity (β = 3.05; 95% CL = 1.52,4.57). When IPCW were excluded, both point estimates and precision did not change in a substantial or consistent way for SCID or PHQ-9 (Appendix Table 6). However, for PHQ-9, models accounting for IPCW resulted in less precise estimates that were further away from the null.

**Table 3:** Maternal ACEs and Depression at 36 Months Postpartum, Bachpan Cohort, Pakistan, *N* = 889

Model		SCID			PHQ-9		
		PR	95% CL		B	95% CL	
**1**	ACE total (DNR = no)	1.20	1.11	1.31	0.65	0.37	0.94
**2**	ACE binary (yes/no)	1.66	1.09	2.53	1.04	0.22	1.86
**3**	ACE 2+	1.62	1.13	2.31	0.99	0.20	1.77
**4**	ACE 3+	1.99	1.32	3.02	2.15	0.99	3.32
**5**	ACE 4+	2.48	1.39	4.41	3.97	2.17	5.77
**6**^**a**^	Ace 1	1.44	0.86	2.40	0.74	−0.40	1.88
	Ace 2	1.33	0.71	2.49	0.15	− 0.76	1.07
	Ace 3	1.91	1.02	3.55	1.09	−0.44	2.62
	Ace 4+	3.13	1.73	5.65	4.37	2.60	6.13
**7**	Neglect	0.89	0.61	1.30	−0.16	−1.24	0.92
**8**	Psychological distress	1.74	1.10	2.75	2.06	0.85	3.26
**9**	Home Violence	1.37	0.97	1.95	0.85	0.25	1.45
**10**	Community violence	2.06	1.29	3.29	3.05	1.52	4.57

Models account for clustering using cluster robust standard errors, and used weights which were a combination of sample weights and inverse probability of censoring weights. All models were adjusted for age, education, and mental health problems in natal family as confounders, and adjusted for trial arm and assessor

*Abbreviations*: *DNR* Does Not Remember, *PR* Prevalence ratio, *B* Estimate, *P p*-value, *CL* Confidence Limit, *ACE* Adverse Childhood Experiences

^a^In Model 6, 0 ACEs is the reference level

## Discussion

In sum, 58% reported at least one ACE and 7% reported four or more. The most common were physical and emotional abuse and physical neglect. Collective violence, being bullied, having an incarcerated family member, and living with someone that was mentally ill were rare with less than 2% experiencing them. By domain, home violence was most common. ACEs were associated with MDE and symptom severity with those experiencing four or more ACEs having a strong relationship with worse mental health. By domain, psychological distress, home violence, and community violence were associated with MDE and symptom severity.

Prevalence of ACEs in this sample was higher than in most high-income settings [6] but lower than in other LMIC (i.e., Kenya) [6, 33]. High prevalence of both abuse and neglect in childhood signal child maltreatment that can lead to mental health problems in adulthood [8]. Low prevalence of family psychological distress may indicate either that women in this area experience or report less family distress as children than others or that the questions may be leading to some degree of under-reporting. Prior work in Pakistan has found that ACEs may be more difficult to disclose in collectivist cultures such as Pakistan where the role of the natal family remains important through adulthood [13]. It may be necessary to modify the ACE-IQ to better fit this cultural context by conducting cognitive interviewing or focus group testing. For example, one study in South Africa deleted two ACE-IQ items after conducting focus groups that inquired about the items’ cultural relevance [34]. Another reason for the low ACEs prevalence in comparison to other LMIC may be because we removed sexual abuse questions due to concerns about sexual abuse histories being under-reported. If disclosed, sexual abuse could potentially put women at risk from their marital families by implying any sexual experience before marriage even though not consensual. Lastly, it is possible that the prevalence of ACEs is lower than expected in this setting. Prior work has found that in South Asia, women are protected until early adulthood when they rapidly transition to marriage, pregnancy, and childbirth and then experience more adversity [35]. Additionally, the Punjab Province government has pushed to increase girls’ education in recent years, which may further protect them from adversity [36].

Given that we found similarly strong relationships between ACEs and symptom severity and ACEs and MDE, ACEs may contribute to both heightened depressive symptom severity and clinical depressive levels. The positive association between ACEs and depression at 36 months postpartum [37] extends previous work in high-income countries focused on depression within 1 year postpartum and corroborates a recent finding that this relationship still exists at 36 months [9].

Additionally, while we did not find evidence of a linear dose-response relationship, we found that women with four or more ACEs exhibited the highest depressive symptomology. A dose-response relationship between ACEs and adverse health has been observed in high-income settings and other LMIC. This provides support for the theory of toxic stress in which high-level exposure to early life adversity increases risk for poor health throughout life [8, 17, 38]. It is possible that at lower levels of ACEs women can draw from resources that reduce risk for depression, but at higher levels of ACEs, these supportive resources are unavailable or overwhelmed. In support of this postulation, a recent study found increased ACEs to be associated with lower amounts of support from family and friends, and that this support mediated the relationship between ACEs and prenatal depression [39]. Future research should expand on the pathways between ACEs and mental health to leverage them into interventions.

Our findings also indicate that ACE domains have unique relationships with women’s depression. Family psychological distress, community violence, and home violence are related to MDE and symptom severity. Community and home violence were associated with maternal depression in Kenya and family psychological distress was in Canada [10, 33]. These exposures to violence and interpersonal trauma in childhood are known to be associated with poor mental health in adulthood. Yet, our study did not align with previous studies regarding the importance of neglect [33]. It is possible that neglect, related to deprivation, has a different relationship to women’s depression than ACEs closely related to threats (i.e., violence) as deprivation and violence may differentially influence neural pathways [40, 41].

The need for future research that conceptualizes ACEs in different sociocultural contexts is reinforced by discordance about the relationship between specific ACE domains and depression by region. Context is instrumental in determining exposure type and frequency and influences the ways individuals learn to process adverse experiences [42]. Additionally, ACE domains likely affect physical and mental health through diverse mechanisms. For example, some neurologic research has demonstrated that different abuse and maltreatment exposures result in various altered brain structures and pathways [43]. ACE domains varying by context and ACE domains differentially affecting adult mental health deserve attention.

### Strengths and limitations

Our study has several strengths. First, we used standardized measures of depression symptom severity and MDE validated in our target population. Second, it is the first study to examine the associations between ACEs and subsequent mental health in South Asia. Third, by using a DAG framework, we estimated the total effect of ACEs on depression and not control for factors affected by ACEs (i.e., adult SES), which leads to biased estimates, as prior studies have done [11, 17].

Multiple limitations warrant discussion. First, 265 women were not followed up at 36 months postpartum. However, no significant difference was found between those that were censored or not based on baseline depression (PHQ-9 > =10), so selection bias by depression status is unlikely. Additionally, we used IPCW to account for missingness, and our results were not sensitive to including these weights (Appendix Table 6). Second, recall bias is likely as ACEs are assessed as a past event [44]. Specifically, depressed women may be more likely to report ACEs than others to understand their depression, resulting in differential misclassification and measurement error. Third, while women’s education is a proxy for childhood SES, we cannot disregard the possibility of residual confounding by childhood SES. Futhermore, although women’s education may sometimes be temporarly subsequent to ACEs, educational attainment up to the primary level more likely reflects family circumstances than individual educational performance, which could be negatively affected by ACEs. Therefore the risk that education mediates the relationship between ACEs and depression, and should not be adjusted for is low. Fourth, there may be a recency effect in which ACEs experienced closer to the age of 18 are more closely related to mental health, but we are unable to explore this with our data. Lastly, as previously stated, the ACE-IQ may not fully capture child adversity in this context.

## Conclusions

Our findings suggest that interventions aimed at both reducing the occurrence of ACEs and mitigating their deleterious impact would be promising in reducing women’s mental health risk in high adversity settings [23]. These interventions are particularly needed in the current global context of the novel coronavirus pandemic, which poses significant mental health threats, particularly for those that have been exposed to ACEs [45]. Our findings also indicate a need to develop context-specific interventions that prevent ACEs from occurring. Perinatal depression and early childhood parenting interventions can reduce ACEs for the next generation [33]. Among women exposed to ACEs, it is important to mitigate their impact on mental illness in adulthood [33, 46]. Prior work has found trauma-focused cognitive-behavioral therapies to be effective at preventing poor mental health among adults exposed to ACEs though this needs to be explored further in LMIC settings [46]. Our findings signal a need for public health practitioners in LMICs to more broadly recognize and address women’s childhood experiences within mental health interventions. Doing so will ensure that women receive appropriate psychosocial and mental health support that accounts for their lifelong experiences rather than only current adversities.

## Data Availability

The datasets used and/or analysed during the current study are available from the corresponding author on reasonable request.

## Appendix

**Table 4:** ACE Question Definitions

Descriptor	Question
**Neglect**	
Emotional Neglect	Caregiver did not understand your problems/worries; did not know what you were doing with your free time
Physical Neglect	Caregiver did not give you enough food or sending you to school even it could easily been done;too drunk/intoxicated by drugs to take care of you
**Family Psychological Distress**	
Alcohol and/or drug abuser in the household	Lived with a household member who was a problem drinker, alcoholic, or misused street/prescription drugs
Incarcerated household member	Lived with a household member who ever sent to jail or prison
Someone chronically depressed, mentally ill, institutionalized or suicidal	Lived with a household member who was depressed, mentally ill, or suicidal
One or no parents, parental separation or divorce	Parents ever separated/divorced, or father/mother/guardian died
**Home Violence**	
Physical Abuse	You being spanked/ slapped/kicked/ punched/beaten up; or being hit/cut with an object by parents/guardian
Emotional Abuse	You were yelled at/screamed at/sworn at/insulted or humiliated, or were threatened/abandoned, thrown out by parents/guardian
Household member treated violently	Saw or heard a parent/household member being yelled at/screamed at/sworn at/ insulted or humiliated; being slapped/kicked/punched/beaten up; or being hit/cut with an object
**Community Violence**	
Bullying	Being bullied
Community Violence	See or hear someone being beaten up, stabbed, shot, or being threatened with a knife or gun in real life
Collective Violence	Forced to go and live in other place; experienced destrubtion of home due to these events; you or family member or friend killed or beaten up by soldiers, police, gangs, or militia

**Table 5:** Baseline Characteristics in IPCW, Bachpan Cohort, Pakistan, *N* = 889^a^

	Mean	SD
People per Room	2.37	1.90
	**N**	**%**
Living with Child’s Grandmother	620	69.10
Living Children		
First Pregnancy	258	31.45
1–3 Children	556	61.05
4+ Children	75	7.50
Asset Quintiles		
First	169	17.01
Second	177	18.89
Third	187	20.90
Fourth	175	21.14
Fifth	181	22.06
SCID	322	26.83
Trial Arm	436	50.94

^a^N’s are unweighted while %, Mean, and SD are weighted by sampling weights and Inverse Probability of Censoring Weights

**Table 6:** Maternal ACEs and Depression at 36 Months Postpartum, Bachpan Cohort, Pakistan, *N* = 889

Model		SCID				PHQ-9			
		PR	P	95% CL		PR	P	95% CL	
1	ACE total (DNR = no)	1.20	0.00	1.10	1.31	0.64	0.00	0.36	0.91
2	ACE binary (yes/no)	1.69	0.01	1.12	2.56	1.04	0.02	0.22	1.86
3	ACE 2+	1.59	0.01	1.11	2.29	0.93	0.02	0.16	1.69
4	ACE 3+	1.90	0.00	1.25	2.88	2.02	0.00	0.88	3.16
5	ACE 4+	2.37	0.00	1.34	4.20	3.84	0.00	2.09	5.59
6^a^	Ace 1	1.51	0.10	0.92	2.49	0.78	0.18	−0.37	1.93
	Ace 2	1.41	0.27	0.76	2.64	0.21	0.65	−0.71	1.12
	Ace 3	1.87	0.05	1.01	3.45	1.01	0.16	−0.42	2.45
	Ace 4+	3.04	0.00	1.69	5.47	4.25	0.00	2.53	5.97
7	Neglect	0.95	0.77	0.64	1.39	−0.04	0.94	−1.15	1.06
8	Psychological distress	1.77	0.02	1.11	2.80	2.00	0.00	0.79	3.22
9	Home Violence	1.35	0.09	0.95	1.92	0.78	0.01	0.19	1.37
10	Community violence	1.96	0.01	1.24	3.08	2.74	0.00	1.38	4.10

Models account for clustering using cluster robust standard errors, and used sampling weights alone, not inverse probability of censoring weights. All models were adjusted for age, education, and mental health problems in natal family as confounders, and adjusted for trial arm and assessor

*Abbreviations*: *PR* Prevalence ratio, *B* Estimate, *P p*-value, *CI* Confidence Limit, *ACE* Adverse Childhood Experiences

^a^In Model 6, 0 ACEs is the reference level

## Abbreviations

LMIC: Low- and middle-income countries
ACEs: Adverse childhood experiences;
US: United States
ACE-IQ: Adverse childhood experiences-International Questionnaire
PHQ-9: Patient Health Questionnaire;
MDE: Major Depressive Episodes
SCID: Structured Clinical Interview for the Diagnostic and Statistical Manual of Mental Disorders
DAG: Directed Acyclic Graph
SES: Socioeconomic Status
IPCW: Inverse probability of censoring weights
